# Frequency of clinical appointments in subjects with emergent suicidal ideation

**DOI:** 10.1192/j.eurpsy.2022.906

**Published:** 2022-09-01

**Authors:** J. Qian, N. Yasmin, V. Deluca

**Affiliations:** Centre of Addiction and Mental Health, Schizophrenia, Toronto, Canada

**Keywords:** Frequency of clinical appointments, suicidal ideation, schizophrénia

## Abstract

**Introduction:**

Schizophrenia is a psychotic disorder strongly associated with suicidal behaviour up to 20-50 times higher than those in the general population. However, treatments from primary healthcare workers and mental health specialists may improve daily function and increase recovery.

**Objectives:**

Our study aims to investigate if the frequency of interactions with healthcare specialists affects suicidal ideation for patients with schizophrenia.

**Methods:**

84 patients diagnosed with schizophrenia spectrum disorder were recruited from the Centre of Addiction and Mental Health (CAMH) in Toronto, Canada. Patient medical charts were reviewed to determine the number of therapeutic interactions in two periods: up to three months from baseline, and retrospectively 3 months before baseline.

**Results:**

19 patients with worsening suicidal ideation had an average of 5.1 more visits following baseline (SD = 6.94), compared to 64 patients with non-emergent SI had 12.0 more visits following baseline (SD = 18.8).

**Conclusions:**

Patients with worsening suicidal ideation had fewer visits from healthcare professionals as compared to those without worsening suicidal ideation. However, further research is necessary to determine the correlation between healthcare visits and suicidal ideation in this population.

**Disclosure:**

No significant relationships.

